# Identification of *POU1F1* Variants in Vietnamese Patients with Combined Pituitary Hormone Deficiency

**DOI:** 10.3390/ijms26062406

**Published:** 2025-03-07

**Authors:** Ha Thu Nguyen, Khanh Ngoc Nguyen, Tran Minh Dien, Thi Bich Ngoc Can, Thi Thanh Ngan Nguyen, Nguyen Thi Kim Lien, Nguyen Van Tung, Nguyen Thi Xuan, Nguyen Thien Tao, Ngoc Lan Nguyen, Van Khanh Tran, Tran Thi Chi Mai, Van Anh Tran, Huy Hoang Nguyen, Chi Dung Vu

**Affiliations:** 1 Hanoi Medical University, 1st Ton That Tung Street, Hanoi 11521, Vietnam; thuha@nch.gov.vn (H.T.N.); khanhnn@nch.gov.vn (K.N.N.); tranchimai@hmu.edu.vn (T.T.C.M.); vananhbiochem@gmail.com (V.A.T.); 2 Center of Endocrinology, Metabolism, Genetic/Genomics and Molecular Therapy, Vietnam National Children’s Hospital, 18/879 La Thanh, Dong Da, Hanoi 11512, Vietnam; ngocctb@nch.gov.vn; 3 Vietnam National Children’s Hospital, 18/879 La Thanh, Dong Da, Hanoi 11512, Vietnam; dientm@nch.gov.vn; 4 Institute of Genome Research, Vietnam Academy of Science and Technology, 18 Hoang Quoc Viet Street, Cau Giay, Hanoi 10072, Vietnam; nganthanh27@yahoo.com (T.T.N.N.); ntkimlienibt@gmail.com (N.T.K.L.); tungnv53@gmail.com (N.V.T.); xuannt@igr.ac.vn (N.T.X.); nguyenthientao@igr.ac.vn (N.T.T.); 5 Center for Gene and Protein Research, Hanoi Medical University, 1st Ton That Tung Street, Hanoi 11521, Vietnam; nguyenngoclan@hmu.edu.vn (N.L.N.); tranvankhanh@hmu.edu.vn (V.K.T.)

**Keywords:** hypopituitarism, combined pituitary hormone deficiency, *POU1F1*, Vietnamese, c.428G>A (p.Arg143Gln), c.811C>T p.(Arg271Trp), c.557T>G p.(Leu186Arg)

## Abstract

Hypopituitarism is a condition characterized by the deficiency of several hormones produced by the pituitary gland. Genetic factors play an important role. Variants in the *POU1F1* gene are associated with combined pituitary hormone deficiency 1 (CPHD1), which manifests as deficiencies in growth hormone (GH), thyroid-stimulating hormone (TSH), and prolactin (PRL). This study aimed to analyze the phenotype, genotype, treatment, and outcomes of Vietnamese patients with deficiency. Six patients from five unrelated families, initially diagnosed with hypopituitarism, were enrolled in this study. Data on physical characteristics, biochemical tests, treatment, outcomes, and follow-up were collected. Exome sequencing and Sanger sequencing were conducted to identify disease-causing variants in five probands and their families. All six patients exhibited anterior pituitary hypoplasia on brain magnetic resonance imaging and presented with TSH, GH, and PRL deficiencies. Exome sequencing identified three variants in the *POU1F1* gene: c.428G>A p.(Arg143Gln), c.557T>G p.(Leu186Arg), and c.811C>T p.(Arg271Trp). The c.811C>T p.(Arg271Trp) variant was found in three patients, while c.557T>G p.(Leu186Arg) is a novel variant. Based on the ACMG classification, these variants were categorized as likely pathogenic or pathogenic variants. All patients were definitively diagnosed with CPHD1 caused by *POU1F1* variants. All patients received levothyroxine and recombinant human growth hormone (rhGH) replacement therapy, leading to considerable growth. During the first year of treatment, all patients showed excellent growth response, with height increases ranging from 11 to 24 cm. After three years of treatment, two patients achieved normal height. One of the six patients developed scoliosis during treatment, which resolved after a one-year pause in rhGH therapy. Upon resuming treatment, no recurrence of scoliosis was observed. Our findings reveal the importance of early hormone testing and genetic analysis in improving the care and outcomes for patients with combined pituitary hormone deficiency.

## 1. Introduction

The pituitary gland is a bean-shaped endocrine organ located at the base of the skull, behind the nose, and between the ears, comprising three lobes: anterior, posterior, and intermediate [1]. The anterior lobe (adenohypophysis) produces six hormones which are regulated by hypothalamic hormones via the hypophyseal portal system [1,2]. The somatomammotroph type of hormones produces growth hormone (GH), produced by somatotrophs, and prolactin, produced by lactotrophs. The glycoprotein hormone types include thyrotrophs producing thyroid stimulating hormone (TSH) with identical alpha subunits and different beta subunits. Gonadotrophs produce follicle-stimulating hormone (FSH) and luteinizing hormone (LH). Corticotrophs produce adrenocorticotrophic hormone (ACTH). The intermediate lobe (melanothophs), which may be an embryological remnant or absent in humans, releases melanocyte-stimulating hormone (MSH) [1,2]. The posterior lobe (neurohypophysis) stores and releases antidiuretic hormone (ADH or vasopressin) and oxytocin, which are contained in vesicles within the hypothalamic-hypophyseal tract [1,2]. Congenital hypopituitarism is characterized by a partial or complete deficiency of pituitary hormone secretion and occurs in 1 in 4000 to 1 in 8000 live births [3]. If left untreated, it can lead to severe, life-threatening complications [4]. Congenital hypopituitarism includes severe midline developmental disorders, such as septo-optic dysplasia or anencephaly; isolated hypopituitarism; or a combination of hypopituitarism with other congenital abnormalities, including short neck, cerebellar malformations, sensorineural hearing loss, and polydactyly. Congenital hypopituitarism is a deficiency of one or more pituitary hormones due to an event occurring during fetal development and typically found at birth [1,5]. This could be due to genetics, antenatal insult or injury, or could be idiopathic. Additionally, hypopituitarism can be part of various genetic syndromes such as Kallmann syndrome, neurohypophyseal diabetes insipidus, septo-optic dysplasia (SOD), holoprosencephaly, multiple pituitary hormone deficiency (MPHD), and isolated hormone deficiencies [1,6]. The pituitary gland plays a crucial role in regulating endocrine function, and disruptions in its hormone secretion can have profound effects on growth, metabolism, and overall health [6]. Therefore, early diagnosis and appropriate hormone replacement therapy are essential for improving patient outcomes.

Pathogenic variants in genes involved in pituitary development can lead to deficiencies in one or more pituitary hormones. Sanger sequencing has identified the genetic etiology in less than 15% of congenital pituitary hormone deficiency cases [7]. Next-generation sequencing has proven to be a valuable tool, increasing the molecular diagnostic yield in hypopituitarism to as much as 19.1% [8,9]. Currently, 70 genes have been reported to be associated with hypopituitarism [8]. The most common disease-causing gene for combined pituitary hormone deficiency (CPHD) is *PROP1* (Prop paired-like homeobox 1), accounting for up to 55% of CPHD cases [10]. Additional causal genes include *POU1F1* (POU class 1 homeobox 1), *HESX1* (homeobox gene expressed in ES cells 1), *LHX3* (LIM homeobox 3), *LHX4* (LIM homeobox 4), *OTX2* (orthodenticle homeobox 2), *GLI2* (GLI family zinc finger 2), and SOX3 (SRY-box transcription factor 3 [10].

The *POU1F1* gene (OMIM *173110) encodes POU class 1 homeobox 1 (*POU1F1* or Pit-1), a protein of 317 amino acids [3] expressed in the anterior pituitary [11,12]. Mutations in *POU1F1* cause combined pituitary hormone deficiency 1 (CPHD1, MIM #613038), which presents with hypoplasia of the anterior pituitary with multiple pituitary hormone deficiencies, resulting in severe short stature, facial dysmorphism, and poor feeding during infancy [10]. The *POU1F1* gene encodes the transcription factor pou1f1, which plays a critical role in the development and differentiation of somatotrophs, lactotrophs, and thyrotrophs, often leading to deficiencies in GH, prolactin, and TSH, respectively [13]. Variants in *POU1F1* cause CPHD1 in both autosomal dominant and, more commonly, autosomal recessive patterns, depending on the genotype [13]. A systematic phenotype-genotype analysis of 114 patients from 58 studies revealed no significant differences between mutation types [14]. However, patients harboring heterozygous mutations, representing the autosomal dominant form of CPHD1, exhibited significantly higher peak growth hormone (GH) levels and a lower prevalence of anterior pituitary hypoplasia compared to patients with homozygous or compound heterozygous mutations, which are typically inherited in an autosomal recessive manner [14]. To date, more than 41 *POU1F1* pathogenic or likely pathogenic variants have been reported in the ClinVar database (https://www.ncbi.nlm.nih.gov/clinvar/?term=POU1F1, accessed on 30 March 2024), with the majority of these variants located within the POU-specific domain (13 of 41 variants) and the POU-homeo domain (11 of 41 variants).

The primary therapeutic strategy for CPHD is hormone replacement therapy, specifically with growth hormone (GH) and thyroid hormone (thyroxine) [15]. Early initiation of these hormone replacements is critical to promote optimal growth and development in affected children. Recombinant human growth hormone (rhGH) therapy has become a cornerstone in managing GH deficiency. In 1985, the US Food and Drug Administration (FDA) issued the first worldwide regulation for rhGH therapy [16]. Initially approved for treating GH deficiency in children, rhGH therapy has since received approval for eight pediatric indications, including GH deficiency, Prader–Willi syndrome, small for gestational age, Turner syndrome, Noonan syndrome, idiopathic short stature (ISS), chronic renal insufficiency, and *SHOX* gene mutations [17]. The starting dose of rhGH and its adjustments are primarily based on body weight or body surface area, with dosing individualized according to national treatment guidelines [18,19].

Studies from the National Cooperative Growth Study (NCGS) and Pfizer International Growth Database (KIGS) showed that a higher weekly rhGH dose could result in better height outcomes [20]. However, larger studies from France (*n* = 1524), Pharmacia/Pfizer (*n* = 1258), and the Netherlands (*n* = 552) found no significant correlation between rhGH doses and adult height (19). Hence, most treatment guidelines recommend starting rhGH therapy at the lower dose range [21]. Additionally, treatment outcomes of the patients were affected by the age at the start of treatment, height at the initiation of therapy, gender, and the degree of GH deficiency [22,23]. Patients who began rhGH therapy at a younger age demonstrated significantly greater improvements in SDS final height [24]. Patients with a lower pre-treatment height SDS typically exhibited a more pronounced growth response during the first year of therapy and sustain superior long-term growth outcomes compared to those with baseline heights closer to the normal range [25]. A total of 23,163 adverse events were reported in 14.4% of patients, with 3108 events (3.1%) assessed as possibly related to treatment [26]. Discontinuation of rhGH therapy (temporary, permanent, or delayed) due to adverse events occurred in 1.6% of patients, with 0.6% attributed to potentially treatment-related adverse events. The most frequently reported adverse event was headache (0.4%), followed by scoliosis (0.2%). All potential risks should be assessed based on the etiological diagnosis and individualized before initiating rhGH treatment. Treatment guidelines recommend informing patients about potential side effects, such as increased intracranial pressure, progressive scoliosis, femoral epiphyseal slippage due to rapid growth, decreased insulin sensitivity, and reduced endogenous cortisol levels resulting from the effect of rhGH on glucocorticoid metabolism. Importantly, rhGH treatment has not been shown to increase the risk of new malignancies in children without pre-existing risk factors.

Herein, we report six patients with hypopituitarism caused by pathogenic or likely pathogenic variants in the *POU1F1* gene. Three missense variants, c.428G>A (p.Arg143Gln), c.557T>G p.(Leu186Arg), and c.811C>T p.(Arg271Trp), were identified, with c.557T>G p.(Leu186Arg) being a novel variant. Marked clinical improvement occurred with rhGH and levothyroxine (LT4) therapy.

## 2. Results

### 2.1. Clinical Findings

**Figure 1:**
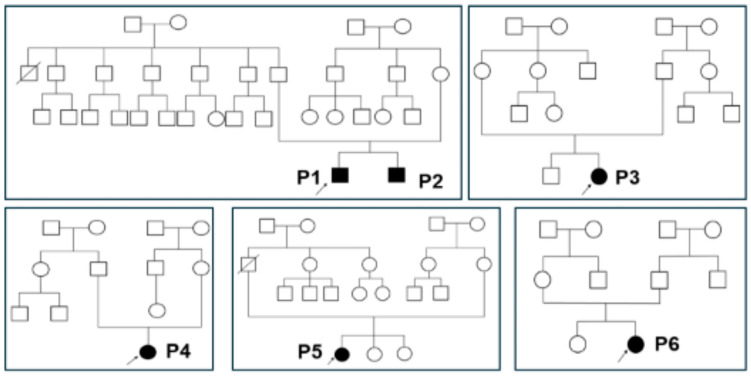
Pedigrees of the five patients’ families in this study. Arrows indicate the probands. Black symbols represent affected patients.

All patients had a normal obstetric history, as well as normal birth weight and height (Table 1). Two patients, P1 and P2, are siblings, while the remaining four patients, P3, P4, P5, and P6, had no family history of CDPH1 (Figure 1). The pedigrees exhibited both autosomal dominant and recessive inheritance patterns (Figure 1). The distribution of traits suggests distinct genetic mechanisms influencing phenotypic expression within the family.

The youngest diagnosis occurred in patient P3 at 20 days old, while the oldest was in patient P4 at 9 years old. Three out of the six patients (P1, P4, and P5) presented with intellectual disability. In contrast, patient P6, diagnosed at eight months old, did not exhibit intellectual disability or motor retardation. All patients showed severe pituitary dwarfism and distinct midfacial hypoplasia, characterized by a prominent forehead, depressed nasal bridge, deep-set eyes, and anteverted nostrils. Bone age was delayed in all patients, with discrepancies between bone age and actual age ranging from six months to eight years.

**Figure 2:**
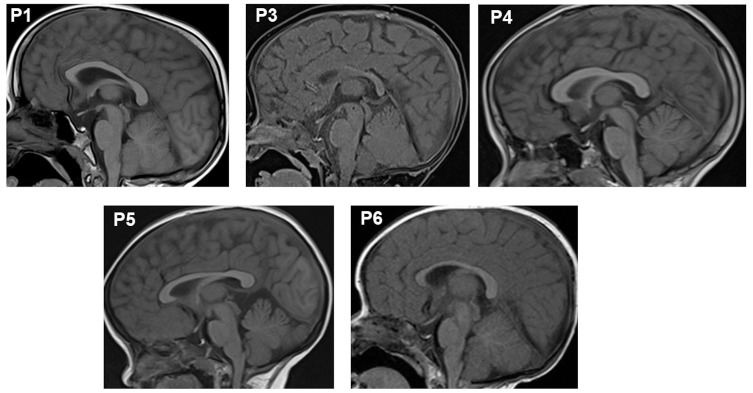
Magnetic resonance imaging (MRI) revealed the anterior hypoplasia in the pituitaries of patients.

Brain magnetic resonance imaging (MRI) revealed anterior pituitary hypoplasia in all six patients (Figure 2). Patient P1 exhibited the most severely hypoplastic anterior pituitary, measuring 1.4 mm, compared to 1.6–1.7 mm in the other patients. The pituitary stalk and posterior pituitary gland were normal in all cases. All patients presented with thyroid hormone and growth hormone deficiencies (Table 1). At the time of diagnosis, P1, P2, and P3 had low levels of thyroid hormones (TSH and T4/FT4). Adrenal function was normal at diagnosis and remained so throughout follow-up. At diagnosis, IGF-1 levels were <15 ng/mL, and GH peaks were ≤0.3 ng/mL. Currently, patient P5 has completed puberty with normal levels of sexual hormones (LH: 4.02 IU/L). All patients exhibited prolactin (PRL) deficiency.

### 2.2. Molecular Findings

**Figure 3:**
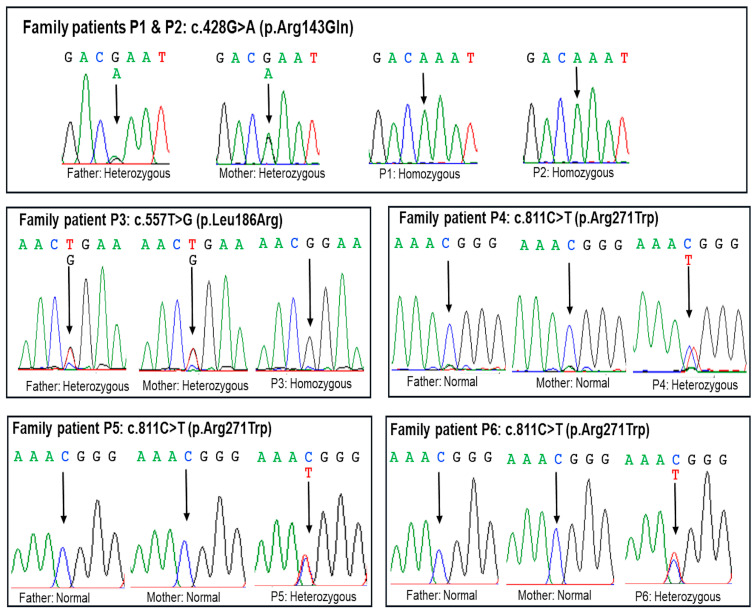
Sanger validation of *POU1F1* variants in the five families. Patients P1 and P2 inherited each mutant allele c.428G>A (p.Arg143Gln) from their parents. Patient 3 was homozygous for c.557T>G p.(Leu186Arg), while her parents were heterozygous. The c.811C>T p.(Arg271Trp) variant was heterozygous in patients P4, P5, and P6. The c.811C>T p.(Arg271Trp) variant was de novo as their parents carried normal alleles.

Exome sequencing was performed, and variants in 70 genes causing CPHD (see the list of genes in Materials and Method) were filtered; the c.428G>A (p.Arg143Gln) variant was identified in patients P1 and P2, the c.557T>G p.(Leu186Arg) variant in patient P3, and the c.811C>T p.(Arg271Trp) variant in patients P4, P5, and P6 in the *POU1F1* gene (Table 2). No pathogenic or likely pathogenic variants were identified in the other CPHD-causing genes. Sanger sequencing was performed to confirm the presence of these variants in the patients and to analyze segregation. The results revealed that patients P1, P2, and P3 inherited the mutant alleles from their parents, suggesting the autosomal recessive mode of inheritance (Figure 3). However, the parents of patients P4, P5, and P6 carried normal alleles, indicating that the heterozygous variant in these patients was de novo, suggesting the autosomal dominant mode of inheritance. The c.557T>G p.(Leu186Arg) variant is a novel variant not reported in the dbSNP154, gnomAD v2.2.1, LOVD v3.0, or ClinVar databases (Table 2). This variant results in the substitution of leucine with arginine at amino acid position 186 and is predicted to have damaging or deleterious effects by multiple in silico tools (Table 2).

Based on ACMG classification, c.428G>A (p.Arg143Gln) and c.557T>G p.(Leu186Arg) variants were classified as likely pathogenic variants with evidence codes PM2, PM3, PP3, and PP4 (Table 2). The c.811C>T p.(Arg271Trp) variant was classified as a pathogenic variant in ACMG guidelines supported by evidence codes PS1, PM1, PM2, PM3, PP3, and PP4 (Table 2).

### 2.3. Outcomes

**Figure 4:**
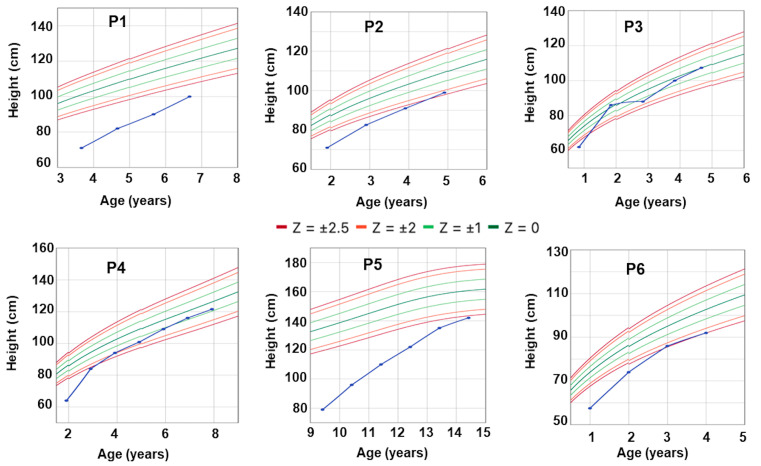
Growth charts of the six patients. The six patients responded well to recombinant human growth hormone (rhGH) therapy. After three years of treatment, patient P1 remained at a low height SDS (−4.37), while patients P2, P3, and P4 reached normal height. Patient P5 reached a height of 143 cm (−2.55 SDS) after five years of treatment. For patient P6, the height has significantly improved, nearing the normal threshold for her age (−2.12 SDS) after two years of treatment.

Five out of the six patients were diagnosed with TSH deficiency and treated with levothyroxine before initiating rhGH therapy. Patient P4 was managed with levothyroxine and rhGH therapy concurrently. The earliest initiation of rhGH therapy was in patient P3 at eight months old, while the latest was in patient P5 at nine years and five months old (Table 3, Figure 4). Patient P5 exhibited severe short stature at the start of rhGH treatment (−9.2 SDS). All patients showed considerable growth improvements with rhGH therapy. In the first year, patients P1 and P2 both grew 11 cm, patients P4, P5, and P6 grew 17–20 cm, and patient P3 achieved a remarkable 24 cm increase (Table 3). After three years of treatment, patients P2, P3, and P4 reached normal height. However, patients P1 and P2 discontinued treatment due to economic constraints. Patient P5 reached a height of 143 cm (−2.55 SDS) after five years of treatment and transitioned to GH therapy during the transition phase. After two years of treatment, P6’s height had significantly improved, nearing the normal threshold for her age (−2.12 SDS). These observations highlight the benefit of initiating rhGH treatment as early as possible to maximize height potential. Other patients continued daily treatment. Patient P3 experienced scoliosis after one year of rhGH therapy, but the condition resolved after nearly one year of discontinuing treatment. Upon resuming therapy, patient P3 did not experience a recurrence of scoliosis.

## 3. Discussion

In this study, we analyzed the phenotype, genotype, treatment, and outcomes of six Vietnamese patients with hypopituitarism. All six patients carried likely pathogenic or pathogenic variants in the *POU1F1* gene. Brain MRI revealed anterior pituitary hypoplasia in all patients, a common feature in *CPHD* patients with *POU1F1* variants [27]. No correlation was observed between pituitary size and patient age or variant type. The patients also exhibited physical characteristics such as midline hypoplasia, upturned nose, and protruding forehead [10,28].

Although all six patients were eventually diagnosed with combined pituitary hormone deficiency (GH, TSH, and PRL deficiencies), achieving an accurate diagnosis in primary care remains challenging. Delayed diagnosis in patients P1, P4, and P5 led to intellectual disability and poor academic performance. In Vietnam, many regions have implemented newborn screening programs, but these primarily involve TSH screening for congenital hypothyroidism. The onset and severity of TSH deficiency varied among patients. For example, patient P4 underwent regular hospital check-ups for elevated liver enzymes starting at 5 months old, but it took over 19 months to diagnose TSH deficiency. Notably, only 13.5% of patients with *POU1F1* variants initially present with isolated GH deficiency, developing additional TSH deficiency after 1–16 years [27]. This shows the importance of long-term monitoring in patients with isolated GH deficiency to detect the progression of additional pituitary hormone deficiencies, particularly in those with *POU1F1* variants. Although PRL deficiency is relatively common, it is rarely tested in Vietnam due to its limited clinical significance in pediatrics. Therefore, we recommend including prolactin testing in the diagnostic workup for patients with GH and TSH deficiencies.

Recurrent hypoglycemia is a common symptom in neonatal patients with severe isolated GH deficiency or combined GH deficiency involving other hormones, especially ACTH [29]. Patients with multiple pituitary hormone deficiencies have a higher incidence of hypoglycemia compared to those with isolated GH deficiency [30]. In our study, none of the patients experienced hypoglycemia, including patient P3, who was diagnosed in the neonatal period. Patients P2 and P3 presented with prolonged neonatal jaundice, a condition observed in 35% of infants with congenital hypopituitarism [31]. This may be explained by GH deficiency affecting liver function through reduced bile acid synthesis and structural abnormalities of the bile duct [31]. Among the patients in this study, only patient P5 had reached puberty (initiating menstruation at 14 years old); the remaining patients in our study have not yet reached the pubertal stage.

**Figure 5:**
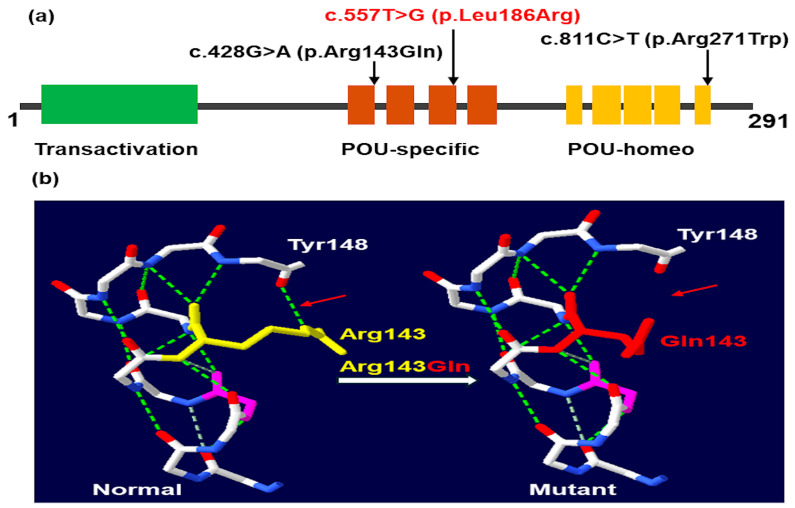
Location of *POU1F1* variants in protein (**a**) and p.Arg143Gln in three-dimensional structure of human PIT-1 (**b**). Arrows indicate the location of the wild-type (**left** side) and mutant (**right** side) amino acid residue.

In this study, we identified two missense variants in the POU-specific domain and one missense variant in the POU-homeo domain of the *POU1F1* gene (Figure 5a). The c.428G>A variant results in an arginine-to-glutamic acid substitution at amino acid position 143, disrupting a hydrogen bond between arginine at position 143 and tyrosine at position 148 (Figure 5b). The c.428G>A (p.Arg143Gln) variant was first reported in a female CPHD patient with severe short stature, normal intellectual ability, and low levels of GH, TSH, and PRL [32]. Our patients P1 and P2 represent the second and third cases with this variant. The phenotype of P1 and P2 closely resembles the case reported by Ohta et al. [32], except for developmental delays in P2, likely due to delayed GH treatment. The c.557T>G p.(Leu186Arg) variant identified in this study is novel. Patient P3, carrying the novel c.557T>G p.(Leu186Arg) variant, was diagnosed in the neonatal period with symptoms including constipation, prolonged neonatal jaundice, pituitary dwarfism, anterior pituitary hypoplasia, and low levels of IGF1, TSH, FT4, GH, and PRL. The third variant, c.811C>T p.(Arg271Trp), was first described by Radovick et al. [33] and has been reported in approximately 30% of patients with *POU1F1* variants [10]. In our study, this variant was identified in three out of the six patients, making it the most common variant in CPHD Vietnamese patients. In this study, TSH levels were higher in patients with heterozygous variants compared to those with homozygous variants. However, we did not observe a correlation between peak GH concentrations, the incidence of anterior pituitary hypoplasia, and the state of the variants, as previously reported (13).

All patients responded well to rhGH replacement therapy, which is consistent with findings from previous studies demonstrating the efficacy of rhGH treatment in CPHD patients. According to the Kabi/Pfizer International Growth Study Database (KIGS), patients with CPHD had a height of –3.8 SDS (–5.8 to –2.3) before treatment, –2.8 SDS (–4.6 to –1.3) after one year, and –1.1 SDS (–3.0 to 0.5) at the subadult stage [34]. Jadhav et al. also reported the clinical efficacy of rhGH in a cohort of CPHD patients with *POU1F1* variants, where 10 out of 15 patients treated with rhGH showed considerable improvement. One patient, treated before one year of age, achieved a final height of –0.9 SDS, highlighting the benefits of early rhGH treatment in achieving nearly normal height [14]. This trend was mirrored in our study, with patient P3, who began treatment at eight months old, achieving normal height within a year. In contrast, patient P5, who started rhGH therapy at nine years and five months, reached a height of 143 cm (–2.55 SDS WHO) at puberty. Additionally, the substantial economic burden of treatment considerably affected compliance, contributing to suboptimal final height outcomes in some patients. CPHD is classified as an intermediate-risk condition in the two largest multicenter longitudinal studies to date: the NordiNet International Outcome Study (2006–2016, Europe) and the ANSWER Program (2002–2016, USA) [35]. The most common adverse event in this risk group is edema, which was not observed in our study population. According to the KIGS database, scoliosis is one of the most frequently reported adverse events (0.2%) during rhGH treatment in patients with GH deficiency or other conditions not associated with CPHD [25]. In our study, scoliosis was observed in only one patient, and this adverse event resolved after treatment resumption.

## 4. Materials and Methods

Six patients from five unrelated families were diagnosed with hypopituitarism based on physical characteristics, biochemical tests, bone age, and brain MRI. The reasons for admission varied among the patients. Patients P1 and P2 were siblings. Patient P1 was admitted at 19 months of age due to intellectual disability. Since two months of age, P1 had experienced slow weight gain, excessive sleep, and severe constipation. Following a diagnosis of central hypothyroidism, the family brought P1’s younger brother (P2) for examination, leading to an early diagnosis at two months of age. Patient P3 was admitted to our department at 20 days old with persistent jaundice. Patient P4 sought medical attention at five months old for slow weight gain and persistently elevated liver enzymes of unknown cause. After more than a year of investigation and hepatitis treatment, P4 was diagnosed with central hypothyroidism and GH deficiency. Patients P5 and P6 were admitted to our hospital due to severe short stature and delayed growth.

Genomic DNA was isolated from peripheral blood according to the manufacturer’s instructions. Exome sequencing was performed on five probands using the Illumina sequencing machine (Illumina, San Diego, CA, USA). The sequences were mapped to the GRCh37/hg19 reference human genome using the Burrows–Wheeler Aligner tool version 0.7.12 [36]. The variant calling was performed using variant calling the Genome Analysis Toolkit version 3.4.0 [37]. The variant annotation was performed using SnpEff version 4.1 g [38]. Pathogenic variants were filtered in 70 hypopituitarism-associated genes, including *ARID1B*, *ARNT2*, *ARID1B*, *ARNT2*, *CHD7*, *FOXA2*, *FOXL2*, *GLI2*, *GLI3*, *GLI4*, *HESX1*, *HMGA2*, *HNF1A*, *LHX3*, *LHX4*, *NFKB2*, *NKX2.1*, *OTX2*, *PAX6*, *POU1F1*, *PROP1*, *RAX*, *SIX1*, *SIX5*, *SIX6*, *SMCHD1*, *SOX2*, *SOX3*, *TCF7L1*, *TGIF1*, *ZIC2*, *ZSWIM6*, *EIF2S3*, *HNRNPU*, *MIR17HG*, *NONO*, *POLR3A*, *RBM28*, *B3GAT3*, *BRAF*, *HID1*, *MAGEL2*, *PCSK1*, *PNPLA6*, *RNPC3*, *SLC15A4*, *SLC20A1*, *SPINK5*, *SPR*, *BMP2*, *BMP4*, *CDON*, *FGF8*, *FGFR1*, *GPR161*, *HHIP*, *IFT172*, *IGSF1*, *IGSF10*, *ANOS1*, *KCNQ1*, *L1CAM*, *LAMB2*, *PROKR2*, *ROBO1*, *SEMA3A*, *SHH*, *TBC1D32*, *TMEM67*, *WDR11*, *NBPF9*, and *BLM* [9]. All variants in the 70 genes with a minor allele frequency ≤0.01 were selected. Intronic variants and synonymous variants were removed. Variants which were predicted as benign variants were eliminated. The pathogenicity of variants was assessed using Mutation Taster [39], and Combined Annotation Dependent Depletion (CADD v1.6) [40]. The pathogenicity of missense variants was further predicted by Sorting Intolerant from Tolerant (SIFT) [41] and Polymorphism Phenotyping v2 (PolyPhen-2) [42]. To confirm the identified variants, exons 3, 4, and 6 of the *POU1F1* gene were amplified using specifically designed oligonucleotide primers, which are available upon request. The PCR products were sequenced using the 3500 Genetic Analyzer capillary electrophoresis system (Life Technologies, Foster City, CA, USA). The reference sequence for *POU1F1* is NM_000306.4. The effect of variants on the three-dimensional structure of human pituitary-specific positive transcription factor 1 (PIT-1; protein data bank code: 5WC9) was modeled using Swiss-PdbViewer v4.1.0 [43].

All patients received levothyroxine and rhGH replacement therapy. Levothyroxine treatment began with an initial dose of 5–10 µg/kg/day, and normal thyroid status was typically achieved within 2–4 weeks. Patients were re-evaluated every 6 months, with dose adjustments based on free thyroxine (FT4) levels. GH therapy was initiated with a dose of 25 µg/kg/day, and the rhGH dose was adjusted every 6–12 months based on serum IGF-1 levels and height velocity.

## 5. Conclusions

Our study identified three pathogenic or likely pathogenic variants in the *POU1F1* gene associated with combined pituitary hormone deficiency in Vietnamese patients. These findings highlight the critical importance of early hormone testing and genetic analysis to improve the diagnosis, management, and outcomes for patients with combined pituitary hormone deficiency.

## Figures and Tables

**Table 1:** Clinical characteristics and hormonal profiles of the six patients.

	P1	P2	P3	P4	P5	P6
**Sociodemographic characteristics**						
Gender	Male	Male	Female	Female	Female	Female
Birth weight (g)	2500	3000	3400	3200	2800	3100
Family history	(+)	(+)	(−)	(−)	(−)	(−)
**Clinical characteristics**						
Age at diagnosis	1y7mo	2mo	Neonate	2y1mo	9y4mo	10mo
Intellectual disability	(+)	(−)	(−)	(+)	(+)	(−)
Pituitary dwarfism	(+)	(+)	(+)	(+)	(+)	(+)
Depressed nasal bridge	(+)	(+)	(+)	(+)	(+)	(+)
Constipation	(+)	(+)	(+)	(+)	(+)	(+)
Umbilical hernia	(+)	(−)	(−)	(−)	(−)	(−)
Prolonged neonatal jaundice	(−)	(+)	(+)	(−)	(−)	(−)
**Pituitary’s MRI**	Anterior hypoplasia	Anterior hypoplasia	Anterior hypoplasia	Anterior hypoplasia	Anterior hypoplasia	Anterior hypoplasia
**Hormonal profiles**						
Peak GH (pmol/L)	<0.30 (≥10)	n/a	0.03 (≥10)	n/a	0.03 (≥10)	n/a
TSH (mU/L)	0.03 (0.70–6.40)	0.01 (1.70–9.10)	0.05 (1.70–9.10)	1.55 (0.70–6.40)	1.00 (0.70–6.40)	1.32 (0.70–6.40)
ACTH (pg/mL) (Normal range)	4.50 (1.60–13.90)	40.16 (1.60–13.90)	4.14 (1.60–13.90)	5.00 (1.60–13.90)	4.74 (1.60–13.90)	51.50 (1.60–13.90)
LH (IU/L) (Normal range)	n/a	n/a	n/a	n/a	4.02 * (0.37–6.23)	n/a
Prolactin (µIU/mL) (Normal range)	<2.00 (63.80–425.00)	<2.00 (63.80–532.00)	2.79 (63.80–532.00)	11.40 (63.80–425.00)	2.38 (63.80–425.00)	5.00 (63.80–532.00)
IGF1 (ng/mL) (Normal range)	<15.00 (33.9–183.9)	<15.00 (11.0–157.0)	7.00 (17.9–125.6)	<15.00 (22.2–145.5)	<7.00 (67.2–349.4)	7.00 (19.5–132.3)
FT4 (pmol/L) (Normal range)	T4: 50.50 (74.00–150.00)	9.60 (14.00–23.00)	0.11 (19.00–39.00)	10.80 (12.00–22.00)	4.24 (12.00–22.00)	4.91 (14.00–23.00)

All hormone tests were performed at the time of diagnosis, except LH of patient P5 (*), which was examined when P5 was 14 years old. Age-specific normal ranges are indicated in parentheses. (+), present; (−), not present; y, year; mo, month; MRI, magnetic resonance imaging; IGF1, insulin-like growth factor 1; TSH, thyroid-stimulating hormone; ACTH, adrenocorticotropic hormone; FT4, free thyroxine; T4, thyroxine; GH, growth hormone; n/a, not analyzed.

**Table 2:** Genetic interpretation of *POU1F1* variants in the six patients.

Patient	P1 and P2	P3	P4, P5, and P6
Gene	*POU1F1*	*POU1F1*	*POU1F1*
Locus	chr3:87313449C>T	chr3:87311268A>C	chr3:87309109G>A
Exon	3	4	6
c.DNA change (NM_000306.4)	c.428G>A	c.557T>G	c.811C>T
Amino acid change	p.(Arg143Gln)	p.(Leu186Arg)	p.(Arg271Trp)
Status in the patient	Homozygous	Homozygous	Heterozygous
Segregation	Paternal and maternal	Paternal and maternal	De novo
Inheritance pattern	Autosomal recessive	Autosomal recessive	Autosomal dominant
CADD (Phred score)	Damaging (33)	Damaging (31)	Damaging (28.7)
SIFT prediction	Deleterious	Deleterious	Deleterious
PolyPhen_2	Probably damaging	Probably damaging	Probably damaging
Mutation Taster	Deleterious	Deleterious	Deleterious
Minor allele frequency	0.000004–0.000008	0	0
dbSNP154	rs104893759	-	rs104893755
ClinVar	13606 Pathogenic	-	13603 Pathogenic
LOVD v3.0	0000886010	-	-
GnomAD v2.1.1	1 Heterozygous	0	0
Pathogenicity (ACMG 2015)	Likely pathogenic PM2, PM3, PP3, PP4	Likely pathogenic PM2, PM3, PP3, PP4	Pathogenic PS2, PM1, PM2, PP3, PP4

CADD, Combined Annotation Dependent Depletion; SIFT, Sorting Intolerant From Tolerant; PolyPhen_2, Polymorphism Phenotyping v2; LOVD, Leiden Open Variation Database; ACMG, American College of Medical Genetics and Genomics; PM, pathogenic moderate; PP, pathogenic support.

**Table 3:** Treatment and responsiveness of the six patients.

	P1	P2	P3	P4	P5	P6
**Age at diagnosis**	1y7mo	2mo	Neonate	2y1mo	9y4mo	10mo
**Age at thyroxine treatment**	1y7mo	2mo	Neonate	2y1mo	9y4mo	10mo
**Age at rhGH treatment**	3y8mo	23mo	8mo	2y1mo	9y5mo	12mo
**Treatment**	Levothyroxin + GH	Levothyroxin + GH	Levothyroxin + GH	Levothyroxin + GH	Levothyroxin + GH	Levothyroxin + GH
✓ Levothyroxin (mg/kg/day)	5–10	5–10	5–10	5–10	5–10	5–10
✓ Initial GH dose (mg/kg/day)	25	25	25	25	25	25
✓ Mean GH dose during treatment (mg/kg/day)	27.5	27.5	25	32.6	22.5	28.3
**IGF1 (ng/mL)**						
✓ After 1 year	44.3	51.5	44.3	74.0	130.0	18.1
✓ After 2 years	7.0	36.6	7.0	76.4	215.0	51.0
✓ After 3 years	7.0	7.0	118.0	161.0	325.0	
✓ After 4 years				147.0	386.0	
✓ After 5 years				38.4	289.0	
**Height (cm) (SDS)**						
✓ Beginning	71.0 (−7.3)	71.0 (−4.8)	62.0 (−3.6)	64.0 (−6.6)	79.0 (−9.2)	57.5 (−6.4)
✓ After 1 year	82.6 (−5.58)	82.0 (−2.69)	86.0 (0.43)	84.0 (−2.9)	96.0 (−6.81)	74.0 (−3.37)
✓ After 2 years	91 (−4.96)	90 (−2.52)	88 (−1.46) *	94 (−2.18)	108 (−5.63)	86 (−2.12)
✓ After 3 years	99 (−4.37)	100 (−1.74)	100 (−0,47)	101 (−2.08)	124 (−4.33)	
✓ After 4 years				108 (−1.94)	135 (−2.85)	
✓ After 5 years				116 (−1.4)	143 (−2.55)	

Y, year; mo, month; rhGH, recombinant human growth hormone; GH, growth hormone. * After 1 year of treatment, patient P3 stopped treatment for one year due to scoliosis.

## Data Availability

The original contributions presented in the study are included in the article; further inquiries can be directed to the corresponding authors.
